# Stability of Spin-Wave Solitons in Bose-Einstein Condensates of Magnons: A Possible Application in Ferromagnetic Films

**DOI:** 10.3390/ma15072551

**Published:** 2022-03-31

**Authors:** Lucas Carvalho Pereira, Valter Aragão do Nascimento

**Affiliations:** 1 Postgraduation Program in Materials Science, Institute of Physics, Federal University of Mato Grosso do Sul, Campo Grande 79070-900, Mato Grosso do Sul, Brazil; 2 Group of Spectroscopy and Bioinformatics Applied to Biodiversity and Health, School of Medicine, Postgraduation Program in Health and Development in the Midwest Region, Faculty of Medicine, Federal University of Mato Grosso do Sul, Campo Grande 79070-900, Mato Grosso do Sul, Brazil; aragao60@hotmail.com

**Keywords:** Bose-Einstein condensates of magnons, Gross-Pitaevskii equation, spin-wave solitons, ferromagnetic films

## Abstract

In this paper, we theoretically investigate the stability of spin-wave solitons in Bose-Einstein condensates of repulsive magnons, confined by an inhomogeneous external magnetic field described by a Gaussian well. For this purpose, we use the quasi-one-dimensional Gross-Pitaevskii equation to describe the behavior of the condensate. In order to solve the Gross-Pitaevskii equation, we used two different approaches: one analytical (variational method) and another numerical (split-step Crank-Nicolson method). The stability of the solutions and the validation of the numerical results were confirmed, respectively, through the anti-VK criterion and the virial theorem. Furthermore, the simulations described the behavior of physical quantities of interest such as chemical potential, energy per magnon and central density as a function of the nonlinearity of the model (magnon-magnon interactions). The theoretical results provide subsidies for a better understanding of the nonlinear phenomena related to the Bose-Einstein condensates of magnons in ferromagnetic films.

## 1. Introduction

Bose-Einstein condensates (BEC), which were experimentally produced in 1995 for alkaline atomic gases of ${}^{87}\mathrm{Rb}$ [1], ${}^{23}\mathrm{Na}$ [2] and ${}^{7}\mathrm{Li}$ [3], provided unique opportunities to investigate macroscopic quantum phenomena in the ultracold temperature regime, both from the point of view of theoretical and experimental physics. Furthermore, recent experiments have demonstrated the possibility of obtaining BECs from quasiparticles, such as excitons [4], polaritons [5] and magnons [6].

Compared to atomic BECs, quasi-particle BECs have two advantages: First, the effective mass of quasiparticles is generally much smaller than the mass of atoms (close to the mass of an electron). This makes the transition temperature higher (in relation to atomic gases) due to inverse proportionality to the effective mass. Second, the density of the quasi-particle system can be increased easily by increasing the external pumping, without worrying about the formation of molecules.

In 1999, Bose-Einstein condensation of magnons was demonstrated in the antiferromagnet ${\mathrm{TlCuCl}}_{3}$ [6] at temperatures around 14K. In 2006, condensation on a yttrium-iron-garnet (YIG) ferromagnetic film—$Y_{3}{\mathrm{Fe}}_{2}O_{12}$—was performed even at room temperature and by means of Brillouin light scattering, they were able to map the density of the magnons as a function of space, frequency, time, and the wave vector on the YIG film, while injecting magnons in an energy state close to that of ferromagnetic resonance by pumping parametric [7]. Since then, BECs of magnons in YIG films have been intensively investigated by Demokritov’s group (Institut für Angewandte Physik (https://www.uni-muenster.de/Physik.AP/Demokritov/Forschen/Forschungsschwerpunkte/mBECfnP.html (accessed on 3 February 2022)). Furthermore, it can be stated that this group is currently demonstrating the formation of BECs of magnons through spin-orbit coupling and the direct spin-Hall effect [8,9,10,11,12].

Recently, Borisenko et al. [10] provided direct experimental evidence that magnon BECs in YIG films are stable with respect to collapse; the origin of this stability being governed by the repulsive magnon-magnon interaction of magnetodipolar nature. However, theoretical studies involving the behavior of chemical potential, energy per magnon, central density, mean square width and nonlinearity coefficient of magnon BECs in YIG films are scarce in the literature.

Therefore, motivated by Ref. [10] and in order to corroborate its results, we propose in this paper, a study on the stability of spin-wave solitons in one-dimensional repulsive magnon BECs trapped by an inhomogeneous external magnetic field, described by a Gaussian well. We will use EGP, within the scope of mean field theory, to describe the BEC of magnons. In order to solve the GPE, we will use two distinct approaches: an analytical approach (variational method) [13,14] and a numerical approach (split-step Crank-Nicolson method) [14,15].

## 2. The Model

In this paper, we propose a scenario where a one-dimensional BEC of magnons, trapped by an external potential, can be described, within the scope of mean field theory, by the following one-dimensional Gross-Pitaevskii equation (GPE):

(1) $$i\hbar \frac{\partial \psi }{\partial t}=-\frac{\hbar^{2}}{2m}\frac{\partial^{2}\psi }{\partial x^{2}}+V_{\mathrm{ext}}\psi +\Gamma {\left|\psi \right|}^{2}\psi .$$

Here, $i=\sqrt{-1}$ is the imaginary unit, ℏ=h/2π is the reduced Planck constant, *m* is the effective mass of each magnon, Γ is the magnitude of the magnon-magnon interaction, $V_{\mathrm{ext}}\equiv V_{\mathrm{ext}}\left(x\right)$ is the trapping potential, determined by an inhomogeneous magnetic field given by $V_{\mathrm{ext}}=g\mu_{B}H$, where *g* is the Landé factor, $\mu_{B}$ is the Bohr magneton and $H\equiv H\left(x\right)$ describes the profile of the inhomogeneous magnetic field. The quantity $\psi \equiv \psi \left(x,t\right)$ is the wavefunction referring to the BEC and is normalized to the total number of magnos:

(2) $$\int {\left|\psi \right|}^{2}dx=N.$$

Moreover, magnon-magnon interactions will be considered repulsive (Γ>0) and the inhomogeneous magnetic field profile described by a Gaussian well:

(3) $$H\left(x\right)=-H_{0}e^{-x^{2}/\nu^{2}},$$

where $H_{0}$ and ν is the amplitude and width of the inhomogeneous magnetic field, respectively.

The use of the one-dimensional GPE is justified by the fact that the cubic nonlinearity in this equation causes solutions of the solitons type. Furthermore, it has been shown that nonlinear dynamical behaviors of spin-wave solitons in ferromagnetic films can be described by a nonlinear Schrödinger equation whose nonlinearity is derived within the scope of the dipole exchange spin-wave spectrum theory [16,17,18]; and a theoretical description of the coherent state of magnons emerging in YIG films for sufficiently strong microwave pumping required an extension of the usual “S-theory” including the GPE for the expected values referring to the magnon operators [19].

Equation (1) can be rewritten using the following dimensionless variables:

(4) $$x\equiv \nu \tilde{x},\;t\equiv \frac{m\nu^{2}}{\hbar }\tilde{t},\;\psi \equiv \sqrt{\frac{N}{\nu }}\tilde{\psi }.$$

Thus, the dimensionless Equation (1) that governs the proposed model becomes (The tilde $\left(\tilde{\;\;\;}\right)$ has been omitted from dimensionless variables to simplify notation.)

(5) $$i\frac{\partial \psi }{\partial t}=-\frac{1}{2}\frac{\partial^{2}\psi }{\partial x^{2}}-V_{0}e^{-x^{2}}\psi +\eta {\left|\psi \right|}^{2}\psi ,$$

where

(6) $$V_{0}\equiv \frac{m\nu^{2}g\mu_{B}H_{0}}{\hbar^{2}},\;\eta \equiv \frac{m\nu N}{\hbar^{2}}\Gamma ,$$

and the wave function is normalized to unity:

(7) $$\int {\left|\psi \right|}^{2}dx=1.$$

It’s important to highlight that the dimensionless values obtained in the simulations can be resized through Eqations (4) and (6) so that they have physical meaning and can be compared with possible experimental observations.

## 3. Methodology

The analytical (variational approach) and numerical (split-step Crank-Nicolson discretization) methods proposed in this paper are motivated by the following attributes: (i) the variational approach can demonstrate in a simple and elegant way some behaviors observed in experiments related to Bose-Einstein condensation. In particular, it quite accurately predicts low-energy nonlinear phenomena in both attractive and repulsive condensates [13,20]. (ii) The split-step Crank-Nicolson method is widely used to solve nonlinear partial differential equations like the GPE associated with BECs and provides highly stable and accurate results while also conserving normalization of the wave function at each iteration [21,22].

### 3.1. Variational Formulation

The problem of solving Equation (5) can be considered as a variational problem corresponding to the minimization of the action S:

(8) S=∫∫Ldxdt,

where Equation (5) can be derived from the following Lagrangian density:

(9) $$L=\frac{i}{2}\left(\psi {\dot{\psi }}^{*}-\psi^{*}\dot{\psi }\right)+\frac{1}{2}{\left(\psi^{\prime }\right)}^{2}-V_{0}e^{-x^{2}}\psi^{2}+\frac{\eta }{2}\psi^{4}.$$

through the Euler-Lagrange equation:

(10) $$\frac{\partial L}{\partial \psi^{*}}-\frac{\partial }{\partial t}\left(\frac{\partial L}{\partial {\dot{\psi }}^{*}}\right)-\frac{\partial }{\partial x}\left(\frac{\partial L}{\partial \psi^{\prime *}}\right)=0,$$

where $\psi^{*}$ is the conjugate complex of ψ, and the quantities $\psi^{\prime }\equiv \partial \psi /\partial x$ and $\dot{\psi }\equiv \partial \psi /\partial t$ are the spatial and temporal derivatives, respectively. The choice of ansatz format is very important [13]. According to the experimental results reported in Ref. [10], the magnon density profile is similar to a Gaussian one. So, we opted for the Gaussian ansatz:

(11) $$\psi \left(x,t\right)=\sqrt{\frac{M}{\pi^{1/2}\sigma }}e^{-x^{2}/2\sigma^{2}}e^{-i\mu t},$$

where μ is the chemical potential and both the norm *M* and the width σ are variational parameters.

Now, our intention is to find the Euler-Lagrange equations that govern the evolution of the variational parameters. For this purpose, we calculate the effective Lagrangian *L* by averaging the Lagrangian density $\langle L\rangle$:

(12) $$L=\langle L\rangle =\int Ldx=\int \left[\frac{i}{2}\left(\psi {\dot{\psi }}^{*}-\psi^{*}\dot{\psi }\right)+\frac{1}{2}{\left(\psi^{\prime }\right)}^{2}-V_{0}e^{-x^{2}}\psi^{2}+\frac{\eta }{2}\psi^{4}\right]dx.$$

where the chemical potential μ was introduced to ensure that the parameter *M* maintains the correct normalization of the wave function ψ. Thus, replacing Equation (11) in Equation (12), the following effective Lagrangian is obtained:

(13) $$L=\left(1-M\right)\mu +\frac{M}{4\sigma^{2}}-\frac{MV_{0}}{\sqrt{1+\sigma^{2}}}+\frac{\eta M^{2}}{2\sqrt{2\pi }\sigma }.$$

The Euler-Lagrange equations for the variational parameters can be obtained via

(14) $$\frac{\partial L}{\partial q}=0,$$

where *q* are the generalized coordinates $q\equiv \left\{\mu ,\sigma ,M\right\}$. The first variational equation, ∂L/∂μ=0, recovers the unit normalization, that is, M=1; which is substituted in the other equations below, except for the equation ∂L/∂M=0, where M=1 is substituted after differentiation. The other equations, ∂L/∂σ=0 and ∂L/∂M=0, yield a set of coupled nonlinear equations:

(15) $$\begin{array}{cc} \;\;\;\;\;\;0 & =\frac{1}{2\sigma^{2}}-\frac{V_{0}\sigma }{\sqrt{1+\sigma^{2}}}+\frac{V_{0}}{{\left(1+\sigma^{2}\right)}^{3/2}}+\frac{\eta }{2\sqrt{2\pi }\sigma }, \end{array}$$

(16) $$\begin{array}{cc} \mu & =\frac{1}{4\sigma^{2}}-\frac{V_{0}}{\sqrt{1+\sigma^{2}}}+\frac{\eta }{\sqrt{2\pi }\sigma }. \end{array}$$

### 3.2. Numerical Discretization

In order to confirm the predictions of variational results, we also searched for numerical solutions referring to Equation (5) through the split-step Crank-Nicolson method. First, Equation (5) is discretized in space and time using the finite difference method [23]. The idea is to divide the spatial (temporal) domain $x_{\mathrm{inicial}}\leq x\leq x_{\mathrm{final}}$$\left(t_{\mathrm{inicial}}\leq t\leq t_{\mathrm{final}}\right)$ into m+1$\left(n+1\right)$ points equally spaced by a spatial (temporal) step $\Delta x=\frac{x_{m}-x_{0}}{m}$$\left(\Delta t=\frac{t_{n}-t_{0}}{n}\right)$, where $x_{0}\equiv x_{\mathrm{inicial}}$ and $x_{m}\equiv x_{\mathrm{final}}$ ($t_{0}\equiv t_{\mathrm{inicial}}$ e $t_{n}\equiv t_{\mathrm{final}}$). Consequently, a mesh consisting of $\left(m+1\right)\times \left(n+1\right)$ points is formed. In general, any point $\left(x_{j},t_{k}\right)$ can be obtained via $x_{j}=x_{0}+j\Delta x$ and $t_{k}=t_{0}+k\Delta t$, j=0,1,…,m and k=0,1,…,n.

The algorithm for the split-step Crank-Nicolson method with imaginary temporal evolution and wave function renormalization can be summarized below [15]:

(17) $$\psi_{j}^{k+1/3}\leftarrow \exp\left[\left(V_{0}e^{-x_{j}^{2}}-\eta_{k}{\left|\psi_{j}^{k}\right|}^{2}\right)\frac{\Delta t}{2}\right]\psi_{j}^{k};$$

(18) $$\psi_{j}^{k+2/3}\leftarrow \psi_{j}^{k+1/3}+\Lambda \left[\left(\psi_{j+1}^{k+2/3}-2\psi_{j}^{k+2/3}+\psi_{j-1}^{k+2/3}\right)+\left(\psi_{j+1}^{k+1/3}-2\psi_{j}^{k+1/3}+\psi_{j-1}^{k+1/3}\right)\right];$$

(19) $$\psi_{j}^{k+1}\leftarrow \exp\left[\left(V_{0}e^{-x_{j}^{2}}-\eta_{k}{\left|\psi_{j}^{k+2/3}\right|}^{2}\right)\frac{\Delta t}{2}\right]\psi_{j}^{k+2/3};$$

(20) $$\psi_{j}^{k+1}\leftarrow \psi_{j}^{k+1}{\left[\int {\left|\psi_{j}^{k+1}\right|}^{2}dx\right]}^{-1/2},$$

where we take $\psi_{j}^{k}\equiv \psi \left(x_{j},t_{k}\right)$ for simplicity of notation and the parameter Λ is given by $\Lambda \equiv \frac{\Delta t}{4{\left(\Delta x\right)}^{2}}$. Furthermore, we use the boundary conditions $\psi_{0}^{k}=\psi_{m}^{k}=0$ to satisfy $\lim_{x\to \pm \infty }\;\psi \left(x,t\right)=0$ and the following initial condition (Gaussian normalized to unity):

(21) $$\psi \left(x,0\right)=\sqrt{\frac{1}{\pi^{1/2}\sigma }}e^{-x^{2}/2\sigma^{2}}.$$

It’s important to note that propagation in imaginary time does not preserve normalization. However, this problem can be overcome by restoring the normalization of the wave function (Equation (20)) after each Crank-Nicolson propagation operation (Equation (18)) [15,24].

Assuming that the wave function is normalized to the unit $\int {\left|\psi \right|}^{2}dx=1$, the chemical potential can be calculated from the following expression:

(22) $$\mu =\int \left[\frac{1}{2}{\left(\frac{d\psi }{dx}\right)}^{2}-V_{0}e^{-x^{2}}\psi^{2}+\eta \psi^{4}\right]dx.$$

The analytical expression for energy per magnon is practically the same as for chemical potential, but with the nonlinear term multiplied by the factor 1/2:

(23) $$\frac{E}{N}=\int \left[\frac{1}{2}{\left(\frac{d\psi }{dx}\right)}^{2}-V_{0}e^{-x^{2}}\psi^{2}+\frac{\eta }{2}\psi^{4}\right]dx,$$

where $E_{\mathrm{kin}}=\int \frac{1}{2}{\left(\frac{d\psi }{dx}\right)}^{2}dx$, $E_{\mathrm{pot}}=\int -V_{0}e^{-x^{2}}\psi^{2}dx$ and $E_{\mathrm{int}}=\int \frac{\eta }{2}\psi^{4}dx$ the kinetic, potential and interaction energies, respectively.

Finally, the parameter $\sigma^{2}$ (mean square width) can be calculated using expression [25]:

(24) $$\sigma^{2}=2\int x^{2}\psi^{2}dx.$$

An important relationship that allows testing the accuracy of the numerical algorithm can be derived through the virial theorem [26,27]:

(25) $$2E_{\mathrm{kin}}+E_{\mathrm{int}}-2V_{0}\int x^{2}e^{-x^{2}}\psi^{2}dx=0.$$

## 4. Results and Discussion

**Figure 1:**
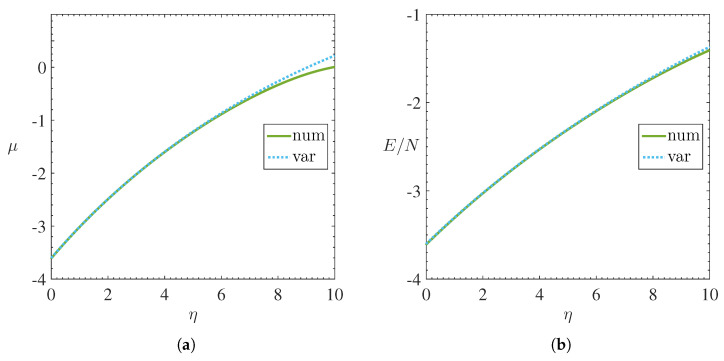
Numerical and variational results illustrating the behavior of (**a**) chemical potential μ and (**b**) energy per magnon E/N as a function of the nonlinearity coefficient η. Solid lines represent numerical solutions while dotted lines represent variational solutions. Stability was confirmed by the anti-VK criterion (dμ/dN>0) observed in (**a**).

**Figure 2:**
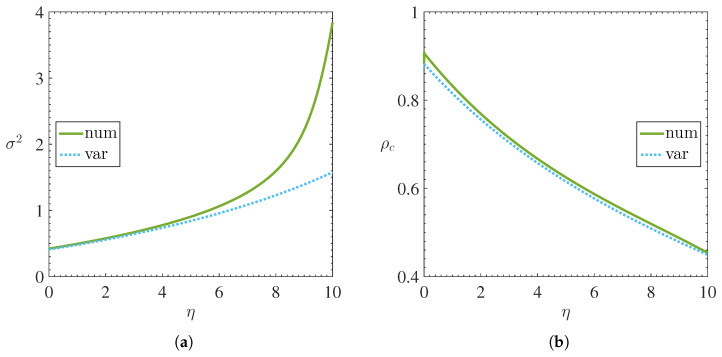
Numerical and variational results illustrating the behavior of (**a**) mean square width $\sigma^{2}$ and (**b**) central density $\rho_{c}$ as a function of the nonlinearity coefficient η. Solid lines represent numerical solutions while dotted lines represent variational solutions.

The Figure 1 and Figure 2 shows, respectively, the behavior of the chemical potential μ, energy per magnon E/N, mean square width $\sigma^{2}$ and central density $\rho_{c}=\lim_{x\to 0}\;{\left|\psi \right|}^{2}$ as a function of nonlinearity η.

Regarding the confining potential, the Gaussian well described very well the confining potential created by a spatially inhomogeneous magnetic field induced by a dc electric current flowing in a control line as proposed by Ref. [10].

**Figure 3:**
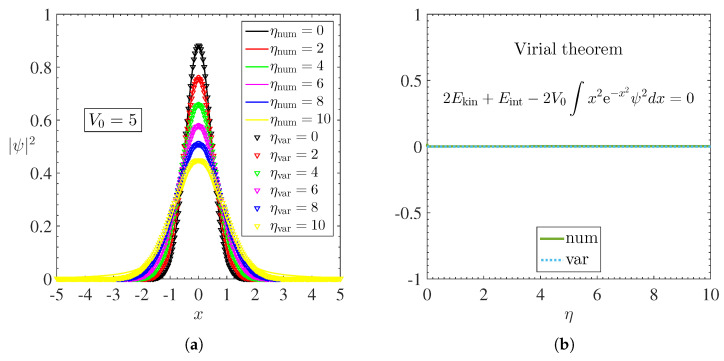
(**a**) Ground states obtained numerically and variationally from Equation (5) for a repulsive BEC of magnons trapped by a Gaussian well for different coefficients of nonlinearity η. Solid lines represent numerical solutions while strings of symbols represent variational solutions. (**b**) Validation of numerical and variational results through the Virial theorem given by Equation (25).

The profiles of the density ${\left|\psi \right|}^{2}$ of the condensate obtained from the numerical solutions (Here, the dimensionless parameters used in the numerical simulations were: $x_{0}=-10$, $x_{m}=10$, $t_{0}=0$, $t_{n}=1000$, Δx=0.02 and Δt=0.005.) (solid lines) and variational (string of symbols) of Equation (5) for a Gaussian well of amplitude $V_{0}=5$ are shown in Figure 3a and, also, illustrates the effects of repulsive nonlinearity on the density distribution: increasing the nonlinearity coefficient η inevitably causes an exponential decay of the peak (central density $\rho_{c}$) and a enlargement of the density ${\left|\psi \right|}^{2}$.

The stability of stationary solutions, both variational and numerical, can be observed in Figure 1a through the anti-VK criterion [28], which says that stable solutions are always found in regions where dμ/dN>0 for repulsive BECs. Furthermore, both the precision and the validation of these results were supported by the Virial theorem, as illustrated in Figure 3b.

Due to the increase in nonlinearity η, the shape of the condensate deviates slightly from the Gaussian. This discrepancy is evident when we compare the mean square width obtained variationally and numerically, according to Figure 2a. Furthermore, this discrepancy was also observed in numerical and variational results obtained in a superfluid Fermi gas model in optical lattice by Adhikari et al. [29].

The results report that any increase in the coefficient of nonlinearity—which corresponds to the density of magnons via Equation (6)—causes an increase in energy per magnon due to magnon-magnon repulsion. Furthermore, we observe that the chemical potential also increases as the nonlinearity increases. Similar behavior was reported in Ref. [7]; increase in chemical potential causing the increase in the density of magnons caused by the microwave pumping technique.

It’s important to emphasize that all the results obtained in this paper describe very well the behaviors that were observed experimentally in BECs of repulsive magnons (η>0) in YIG films created by the Demokritov group and reported in Ref. [10].

## 5. Conclusions

This paper was based on the study of the stability of spin-wave solitons in Bose-Einstein condensates of magnons subjected to repulsive interactions, confined by an inhomogeneous Gaussian-well magnetic field. For this purpose, we use the Gross-Pitaevskii equation to describe the condensate. In order to solve the EGP, we used two different approaches: one analytical (variational method) and another numerical (split-step Crank-Nicolson method). In both approaches, we used the Gaussian function to describe the ansatz, referring to the variational method, and the initial condition, referring to the split-step method. In general, we obtained a reasonable agreement between the variational results and the numerical results related to the EGP associated with the proposed model. Furthermore, the stability of the solutions was verified through the anti-VK criterion and the validation of the results were supported by the virial theorem, in addition to being in accordance with experimental results obtained recently. Finally, we strongly believe that the theoretical results reported in this paper can open doors to better understand nonlinear phenomena referring to Bose-Einstein condensates of magnons in YIG films.

## Data Availability

Not applicable.
